# Comprehensive allostatic load risk index is associated with increased frontal and left parietal white matter hyperintensities in mid-life cognitively healthy adults

**DOI:** 10.1038/s41598-023-49656-3

**Published:** 2024-01-05

**Authors:** Ingrid Buller-Peralta, Sarah Gregory, Audrey Low, Maria-Eleni Dounavi, Katie Bridgeman, Georgios Ntailianis, Brian Lawlor, Lorina Naci, Ivan Koychev, Paresh Malhotra, John T. O’Brien, Craig W. Ritchie, Graciela Muniz-Terrera

**Affiliations:** 1 Edinburgh Dementia Prevention, Centre for Clinical Brain Sciences, Outpatients Department Level 2 Western General Hospital, The University of Edinburgh, Crewe Rd S, Edinburgh, EH4 2XU UK; 2 Department of Psychiatry, School of Clinical Medicine, Addenbrooke’s Hospital, University of Cambridge, Level E4, Box 189, Cambridge, CB2 0QQ UK; 3 Trinity College Institute of Neuroscience, School of Psychology, Aras an Phiarsaigh, Trinity College Dublin, Dublin 2, Ireland; 4 Global Brain Health Institute, Trinity College Dublin, GBHI Office Room 0.60, Lloyd Building Trinity College Dublin, Dublin 2, Ireland; 5 Department of Psychiatry, Warneford Hospital, Oxford University, Warneford Ln, Headington, Oxford, OX3 7JX UK; 6 Department of Brain Sciences, Imperial College London, Burlington Danes, The Hammersmith Hospital, Du Cane Road, London, W12 0NN UK; 7 Scottish Brain Sciences, Gyleview House, 3 Redheughs Rigg, South Gyle, Edinburgh, EH12 9DQ UK; 8 Ohio University Heritage College of Osteopathic Medicine, 191 W Union St, Athens, OH 45701 USA

**Keywords:** Neuroscience, Risk factors

## Abstract

To date, there is a considerable heterogeneity of methods to score Allostatic Load (AL). Here we propose a comprehensive algorithm (ALCS) that integrates commonly used approaches to generate AL risk categories and assess associations to brain structure deterioration. In a cohort of cognitively normal mid-life adults (n = 620, age 51.3 ± 5.48 years), we developed a comprehensive composite for AL scoring incorporating gender and age differences, high quartile approach, clinical reference values, and current medications, to then generate AL risk categories. Compared to the empirical approach (ALES), ALCS showed better model fit criteria and a strong association with age and sex. ALSC categories were regressed against brain and white matter hyperintensity (WMH) volumes. Higher AL risk categories were associated with increased total, periventricular, frontal, and left parietal WMH volumes, also showing better fit compared to ALES. When cardiovascular biomarkers were removed from the ALSC algorithm, only left-frontal WMHV remained associated with AL, revealing a strong vascular burden influencing the index. Our results agree with previous evidence and suggest that sustained stress exposure enhances brain deterioration in mid-life adults. Showing better fit than ALES, our comprehensive algorithm can provide a more accurate AL estimation to explore how stress exposure enhances age-related health decline.

## Introduction

Allostatic Load (AL) describes the wear and tear resulting from physiological responses upon chronic stress exposure^1,2^. The first AL index described included neuroendocrine, metabolic, immune, and cardiovascular biomarkers, classified in two categories: primary mediators, like cortisol or epinephrine, released as first response of the hypothalamic-pituitary axis (HPA) upon stress exposure; and secondary outcomes, or downstream responses activated by primary mediators, such as glycemia or blood pressure. For scoring biomarkers, higher quartile values based on sample distribution were used to define risk, and then summed into a total AL score^3^. Novel approaches have excluded primary mediators, focusing on cardiovascular, metabolic, and inflammatory markers, which are easier to assess and collect in large cohorts. Nowadays, there is considerable heterogeneity in AL scoring methods. McLoughlin et al., compared 14 different algorithms and concluded that the association of AL with health outcomes is robust to variations in the method^4^. However, they also reported that quartiles calculation based on sex-specific distributions and inclusion of prescribed medications provide a strong improvement in model fit relative to the purely empirical quartile-based algorithm. Current evidence favour more comprehensive approaches that include prescribed medications and clinical thresholds when available^5,6^, and the need to consider sex differences among biomarkers is strongly supported by both clinical practice^7,8^ and research evidence^9–11^.

Accordingly, we propose a comprehensive composite for AL scoring (ALCS) that includes sex and age differences, a high-quartile approach, clinical thresholds, and current medications, to develop a more accurate index and generate AL risk categories.

To date, evidence reports that several demographic and health covariates are predicted by high AL, such us males over females, increased age, fewer years of education, low income, smoking, poor sleep quality, APOEe4 carriers, and dementia risk^5,9,11–13^. Thus, after statistical validation of the ALCS algorithm, these covariates were assessed for replicability on the AL risk categories.

Additionally, AL has been associated with increased risk of cognitive impairment^14^, Alzheimer's disease (AD)^13^, and brain deterioration^15–17^. However, evidence related to brain changes in senior participants (> 60 years) remains diverse, with some showing strong negative associations to grey but not white-matter volumes^16,17^, and others reporting lower white-matter and hippocampal volumes^15^. As all these studies derived an AL scoring using a purely empirical approach, we evaluated whether our ALCS risk categories could replicate previous findings and better predict volumetric changes and white matter hyperintensities (WMH) across several brain areas.

## Results

### Participants

The analytical sample included 620 participants from the PREVENT dementia study (61.13% females), with an average age of 51.3 (SD = 5.48) years old (females: 50.97, SD = 5.41), and a mean of 16.62 (SD = 3.44) years of education. Demographic characteristics of the PREVENT participants included the analyses are detailed in Table 1. No differences were found between males and females for age and years of education (Supplementary Table S1).

**Table 1:** Demographic characteristics of the studied population.

Variable [missing]			
Sex, n (%)		Male	241 (38.87%)
		Female	379 (61.13%)
		Total	Females
Age, mean (SD)	Total range 40–60 (IQR = 9)	51.26 (5.48)	50.97 (5.41)
Years of education, mean (SD) [1]		16.62 (3.44)	16.79 (3.58)
Education, n (%) [3]	Less than high school	2 (0.32%)	0 (0%)
	High school / Secondary school	93 (15.07%)	58 (15.34%)
	College / University	280 (45.38%)	165 (43.65%)
	Trade / Technical / Non-University	64 (10.37%)	38 (10.05%)
	Post-graduate	178 (28.85%)	117 (30.95%)
Employment, n (%)	No current employment	87 (14.03%)	55 (14.51%)
	Employed	533 (85.97%)	324 (85.49%)
Smoking History, n (%) [1]	Current	34 (5.49%)	17 (4.5%)
	Past	228 (36.83%)	137 (36.24%)
	Never	357 (57.67%)	224 (59.26%)
Dementia Risk, n (%)	Parent dementia History	314 (50.65%)	200 (52.77%)
	Direct relative Alzheimer's disease	137 (22.1%)	88 (23.22%)
	APOEe4 carriers	235 (38.21%)	142 (60.43%)
Subjective Memory Complaint, n (%)		182 (29.35%)	116 (30.61%)
ALCS risk categories, n (%)	No risk	14 (2.26%)	13 (3.43%)
	Low risk	191 (30.81%)	114 (30.08%)
	Medium risk	230 (37.09%)	136 (35.88%)
	High risk	185 (29.84%)	116 (30.61%)
Pittsburgh Sleep Quality Index Questionnaire (PSQI)	PSQI Global Score, Mean (SD)	6.03 (3.07)	6.16 (3.1)
Connor-Davidson Resilience Scale (CDRS)	Total Score, Mean (SD)	72.83 (12.98)	72.33 (13.31)

### Comprehensive AL (ALCS) algorithm validation

**Figure 1:**
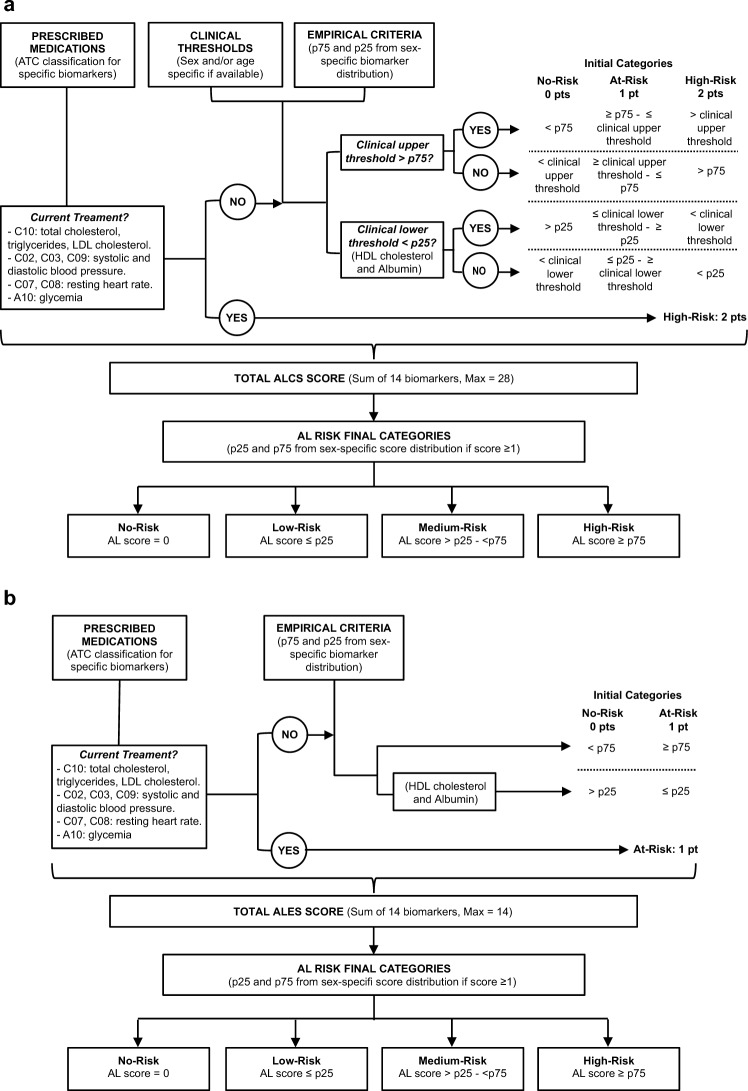
Algorithm flowchart for AL scoring and AL risk categories construction. (**a**) Algorithm for comprehensive (ALCS) and (**b**) empirical (ALES) scores construction.

The comprehensive AL score (ALCS) and the empirical AL score (ALES) were calculated following the algorithm flowchart described in Fig. 1, to create AL risk categories. Final risk categories of No-risk, Low-Risk, Medium-risk and High-risk were created for both algorithms.

Each categorical output was regressed separately for model fit comparison. Multinomial logistic regressions (MLR) for AL categories as dependent variable showed satisfactory fitting for both scoring algorithms, with a slight difference between Mc Fadden’s pseudo-R^2^ in favouring the ALCS scoring (ΔR^2^ = 0.013). Nonetheless, ALCS showed higher log-likelihood (ΔLL = 40.747) and substantial decrease of the Bayesian Information Criterion (ΔBIC = − 81.494), compared to ALES. Both algorithms showed significant effects for age (Likelihood ratios χ^2^(3) = 18.35, *p* < 0.001; χ^2^(3) = 29.24, *p* < 0.001) and education (Likelihood ratios χ^2^(3) = 11.96, *p* = 0.008; χ^2^(3) = 13.93, *p* = 0.003), but only ALCS displayed a significant effect for sex (Likelihood ratios for ALCS χ^2^(3) = 8.28, *p* = 0.041, versus ALES χ^2^(3) = 2.16, *p* = 0.541).

Analysis between the ratios of correct classification into AL categories showed no significant differences among algorithms, although low-risk category appeared better ranked in ALCS. Inter-rater reliability test showed a slight level of agreement between algorithms (Cohen’s kappa test for 4 measures and 2 scorers: κ = 0.147) however, z test revealed a significant relation between scorers (z = 5.033, *p* < 0.001). Finally, both algorithms were correlated against age to assess sensitivity. Both algorithms showed significant positive Spearman's correlation coefficients (ALES r_s_ = 0.172, *p* < 0.001; ALCS r_s_ = 0.201, *p* < 0.001) and no significant differences between coefficients were found after Fisher’s z transformation (z = 0.528, *p* = 0.298). Table 2 shows model fit comparisons between ALCS and ALES algorithms. Overall, even when no significant differences were found between the standard algorithm based on high-quartiles and our comprehensive composite, the latter proved better model fit criteria.

**Table 2:** Model fit comparison of comprehensive (ALCS) and empirical (ALES) algorithms.

Model fit statistics (AL category = Sex + Age + Years of education)							
	Model Fitting	Pseudo R^2^	ΔR^2^	BIC	ΔBIC	Log-likelihood (LL)	Δ LL
ALES	χ^2^ (9) = 35.666, * p* < 0.001	0.023	REF	1237.12	REF	− 579.9815	REF
ALCS	χ^2^ (9) = 53.241, * p* < 0.001	0.036	0.013	1155.63	− 81.494^a^	− 539.2345	40.747

Rate of correct classification							
		No-risk	Low-risk	Medium-risk	High-risk	Overall	
ALES	observed	35	181	246	158	620	
	predicted	0	14	211	29	254	
	% correct	0	7.7	85.8	18.4	41	
ALCS	observed	14	191	230	185	620	
	predicted	0	71	133	57	261	
	% correct	0	37.2	57.8	30.8	42.1	
p̂ Comparisons	pooled p̂	0	0.228	0.723	0.251	0.415	
	SEDp	0	0.123	0.05	0.099	0.043	
	z	0	2.398	5.638	1.26	0.26	
	p (α = 0.05)	0.5	0.008	< 0.001	0.104	0.397	

Inter-rater reliability							
Agreement	p Error	Cohen's κ	SEκ	95% CI		Z transformed	p
0.776	0.314	0.147	0.029	0.09	0.204	5.033	< 0.001

Correlation AL category versus Chronological Age							
	Spearman's rho (r_s_)	*p*	Z	Δ Z	Observed Z	*p* (α = 0.05)	
ALES	0.172	< 0.001	0.174	REF			
ALCS	0.201	< 0.001	0.204	0.03	0.528	0.298	

BIC, Bayesian Information Criterion; BIC Δ, Difference in BIC compared with the classic empirical AL scoring algorithm.

^a^Substantial decrease in BIC compared with the classic empirical AL scoring algorithm.

### Associations between ALCS and demographic variables

Associations between ALCS risk categories and demographic covariates were estimated through MLR (Supplementary Table S2). The model showed satisfactory fitting (χ^2^(27) = 64.78, *p* < 0.001), with significant effects for age (χ^2^(3) = 24.66, *p* < 0.001), sex (χ^2^(3) = 9.05, *p* = 0.029), and educational level (χ^2^(3) = 9.89, *p* = 0.019). Age was positively associated with AL, with significantly increased odds of being in medium (aOR = 1.16, *p* = 0.009) and high-risk AL (aOR = 1.2, *p* = 0.002) respectively). Conversely, sex showed that males had significantly higher odds for being at low (aOR = 0.11, *p* = 0.035) and medium-risk (aOR = 0.11, *p* = 0.034) AL categories. No significant associations were found between AL risk and educational level, employment status, smoking history, parental history of dementia, direct relatives with AD (parents or siblings), APOEe4 carrier status, or subjective memory complaints.

### Associations between ALCS, brain volume and white matter hyperintensity volumes (WMHV)

571 Participants (females: n = 344) had MRI at baseline for white-matter, total grey-matter, subcortical grey-matter, left and right hippocampal volume measurements (WM, GM, SCG, lHC, rHC, respectively). 481 participants (females: n = 291) had data for CA1 volume, and 566 (females: n = 341) for WMH analysis. Greater raw and adjusted volumes after residual correction were found in men, however hippocampal and CA1 values were greater in females after adjustment (Supplementary Table S3).

To evaluate associations between ALCS risk categories and brain volumetric measurements, a hierarchical linear approach with three blocks of variables was used. First, we considered a univariate regression between volume measurement and ALCS categories. Second, for a partially adjusted model, we included the same demographic variables used for validating the ALCS (sex, age and years of education). Finally, a fully adjusted third model included the Pittsburgh Sleep Quality Index (PSQI) global score and the Connor-Davidson Resilience Scales (CDRS) score as additional covariates potentially affected by stress exposure.

A first set of regressions was conducted for WM, GM, SCG, lHC, rHC, CA1 and total WMHV (Table 3). The univariate model showed no association between AL categories and volumetric measurements, except for total WMHV (F(1,565) = 7.7, *p* = 0.005). The partially and fully adjusted models provided better fit in all the regressed variables, with exception of the lHC and rHC volumes, although no significant associations emerged.

**Table 3:** Hierarchical regression analysis of adjusted brain volumes cortical thickness and total WMHV.

		WM vol^a^	GM vol^a^	SCG vol^a^	SCG vol^a^ ǂ	lHC vol^a^	rHC vol^a^	CA1 vol^b^	LH thickness	RH thickness	Total WMHV^c^
Model 1	ALCS	0.011	− 0.029	0.059	0.059	0.005	− 0.032	0.075	− 0.026	− 0.022	0.116**
	R^2^	0.000	0.001	0.003	0.003	0.000	0.001	0.006	0.001	0.000	0.013
	F (1,570)	0.07	0.48	1.97	F(1,570) 1.97***	0.02	0.6	F(1,480) 2.74	0.38	0.28	F(1,565) 7.7**
Model 2	ALCS	0.022	0.015	[0.103*]	0.061, * p* = 0.143	0.016	− 0.021	0.083	0.016	0.018	0.083*
	Sex	− 0.137**	− 0.28***	− 0.187***	− 0.173***	0.080	0.068	0.231***	0.054	0.045	− 0.140***
	Age	− 0.035	− 0.145***	− 0.223***	***excluded ǂ***	− 0.067	− 0.041	− 0.021	− 0.162***	− 0.153***	0.153***
	Years Ed	0.043	0.14**	0.012	0.033	− 0.029	0.022	− 0.010	0.072	0.069	− 0.022
	R^2^	0.021	0.112	0.081	0.034	0.012	0.009	0.060	0.038	0.033	0.060
	F (4,570)	2.99*	7.87***	2.45***	F(3,570) 6.59***	1.67	1.22	F(4,480) 7.56***	5.56***	4.84**	F(4,565) 0.89***
Model 3	ALCS	0.024	0.018	[0.113**]	0.07, * p* = 0.094	0.015	− 0.020	0.084	0.007	0.007	0.09*
	Sex	− 0.134**	− 0.279***	− 0.181***	− 0.167***	0.081	0.070	0.234***	0.048	0.038	− 0.138***
	Age	− 0.036	− 0.146***	− 0.225***	***excluded ǂ***	− 0.068	− 0.042	− 0.020	− 0.16***	− 0.151***	0.152***
	Years Ed	0.043	0.14**	0.014	0.035	− 0.029	0.023	− 0.011	0.070	0.067	− 0.021
	PSQI	0.020	− 0.023	− 0.037	− 0.034	0.025	0.020	0.017	0.022	0.040	− 0.057
	CDRS	0.103*	− 0.009	0.1*	0.098*	0.073	0.079	0.069	− 0.118**	− 0.114**	− 0.008
	R^2^	0.031	0.113	0.094	0.046	0.017	0.014	0.064	0.054	0.050	0.063
	F (6,570)	2.96**	1.93***	9.77***	F(5,570) 5.451***	1.59	1.37	F(6,480) 5.42***	5.33***	4.92***	F(6,565) 6.29***

Standardized regression coefficients reported.

ALCS, Allostatic load comprehensive scoring categories; Years Ed, Years of education; PSQI, Pittsburgh sleep quality index global score; CDRS, Connor-Davidson resilience scale; WM, white matter volume GM, grey matter volume; SCG, subcortical grey matter volume; lHC, left hippocampal volume; rHC, right hippocampal volume; CA1, Hippocampal CA1 subregion volume; LH thickness, left hemisphere mean thickness; RH thickness, right hemisphere mean thickness; WMHV, white matter hyperintensity volume.

^a^Adjusted to eICV by residual correction method.

^b^CA1 adjusted to eTIV by residual correction method.

^c^Normalized to SPM12 total intracranial volume (TIV).

Values between brackets represent artifactually increased associations due to suppressor variable effect.

ǂSuppressor variable age excluded from regression model.

*p < 0.05; **p < 0.01; ***p < 0.001.

No associations were found between AL categories and WM or GM volumes, however greater GM volume was predicted in younger male participants with higher years of education in the partial (sex: β = − 0.280, *p* < 0.001; age: β = − 0.145, *p* < 0.001; education: β = 0.140, *p* = 0.001) and fully adjusted model (sex: β = − 0.279, *p* < 0.001; age: β = 0.146, *p* < 0.001; education: β = 0.140, *p* = 0.001).

Although subcortical grey matter volume (SCG) showed a positive association with AL categories in the partial (β = 0.103, *p* = 0.013) and fully adjusted (β = 0.113, *p* = 0.007) models, the inconsistency with the univariate model suggested an artificially improved regression coefficient due to a suppressor variable effect. Accordingly, when the covariate age was removed from the partially and fully adjusted models, the association between SCG and AL risk category was lost (see Table 3, for SCG volume coefficients between brackets and corrected models), and greater SCG volumes were only found associated with participants with higher resilience scores (β = 0.098, *p* = 0.02).

Finally, total WMHV was positively associated with higher AL risk categories in all models (β = 0.116, *p* = 0.006; β = 0.083, *p* = 0.046; β = 0.09, *p* = 0.035, for the univariate, partial and fully adjusted models, respectively).

A second set of regressions was conducted to evaluate local WMHV in the peri-ventricular (PV), deep, left-frontal (LF) right-frontal (RF), left-parietal (LP), right-parietal (RP), left-occipital (LO), right-occipital (RO), left-temporal (LT), and right-temporal (RT) areas (Table 4). Greater WMHV in the PV, LF and RF regions were predicted by higher AL categories in the univariate regression model (PV: β = 0.135, *p* = 0.001; LF: β = 0.171, *p* < 0.001; RF: β = 0.119, *p* = 0.005), and the association was maintained after including the second and third block of covariates. Increased LP and LT WMHV were only predicted by higher AL in the univariate model (β = 0.086, *p* = 0.041; β = 0.083, *p* = 0.046, respectively). Deep WMHV was not predicted by AL in any of the models fitted.

**Table 4:** Hierarchical regression analysis of WMHV by area.

(Total sample N = 566; females N = 341)											
		PV WMHV	Deep WMHV	LF WMHV	RF WMHV	LP WMHV	RP WMHV	LO WMHV	RO WMHV	LT WMHV	RT WMHV
Model 1	AL	0.135**	0.067	0.171***	0.119**	0.086*	0.014	0.061	0.04	0.083*	0.001
	R^2^	0.018	0.004	0.029	0.014	0.007	0.000	0.004	0.002	0.007	0.000
	F (1,565)	0.54**	2.52	7.06***	8.04**	4.2*	0.11	2.07	0.91	3.97*	0.0001
Model 2	AL	0.095*	0.046	0.141**	0.089*	0.06	− 0.002	0.034	0.003	0.053	− 0.028
	Sex	− 0.125**	− 0.13**	− 0.089*	− 0.089*	− 0.099*	− 0.097*	− 0.276***	− 0.284***	− 0.104*	− 0.093*
	Age	0.182***	0.084	0.14**	0.135**	0.11*	0.059	0.135**	0.174***	0.116**	0.11*
	Years Ed	− 0.021	− 0.018	− 0.015	− 0.018	− 0.026	− 0.029	0.03	0.005	− 0.016	− 0.044
	R^2^	0.071	0.031	0.059	0.042	0.032	0.015	0.102	0.118	0.032	0.025
	F (4,565)	0.66***	4.42**	8.76***	6.18***	4.63**	2.18	5.87***	8.77***	4.7**	3.59**
Model 3	AL	0.102*	0.054	0.147**	0.092*	0.063	0.008	0.046	0.015	0.06	− 0.019
	Sex	− 0.122**	− 0.127**	− 0.087*	− 0.088*	− 0.099*	− 0.093*	− 0.272***	− 0.28***	− 0.102*	− 0.09*
	Age	0.182***	0.083	0.14**	0.134**	0.11*	0.058	0.133**	0.172***	0.116**	0.109*
	Years Ed	− 0.02	− 0.017	− 0.014	− 0.018	− 0.026	− 0.028	0.032	0.007	− 0.015	− 0.043
	PSQI	− 0.051	− 0.052	− 0.038	− 0.021	− 0.045	− 0.068	− 0.067	− 0.079	− 0.072	− 0.064
	CDRS	− 0.013	0.001	0.004	0.005	− 0.067	− 0.001	0.027	0.001	− 0.067	− 0.01
	R^2^	0.073	0.033	0.060	0.043	0.037	0.020	0.108	0.124	0.040	0.029
	F (6,565)	7.34***	3.2**	5.98***	4.16***	3.58**	1.88	1.24***	3.21***	3.84**	2.76*

WMHV normalized to SPM12 total intracranial volume (TIV).

Standardized regression coefficients reported.

WMHV, white matter hyperintensity volume; AL, Allostatic load category; Years Ed, Years of education; PSQI, Pittsburgh sleep quality index global score; CDRS, Connor-Davidson resilience scale; PV, peri-ventricular; LF, left-frontal; RF, right-frontal; LP, left-parietal; RP, right-parietal; LO, left-occipital; RO, right-occipital; LT, left-temporal; RT, right temporal.

*p < 0.05; **p < 0.01; ***p < 0.001.

**Figure 2:**
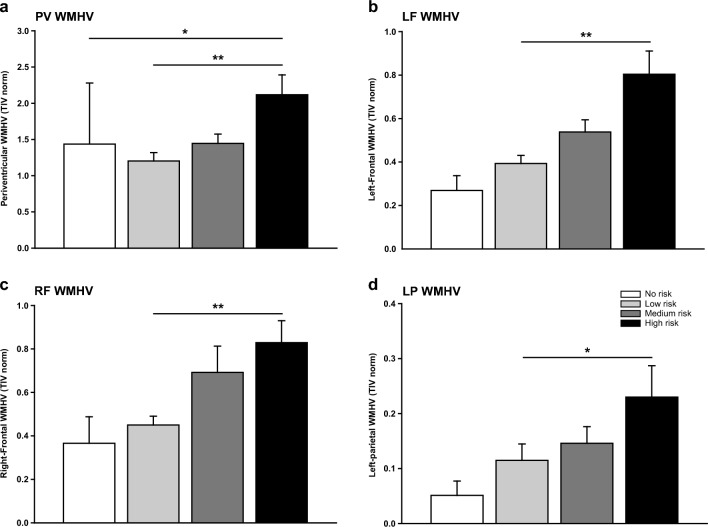
Individuals within high-risk ALCS category show increased white matter hyperintensity volume. Comparison between AL categories and (**a**) periventricular, (**b**) left frontal, (**c**) right frontal and (**d**) left parietal WMHV. Kruskal–Wallis test for independent groups, followed by a Bonferroni corrected Dunn’s test for multiple comparisons (*p < 0.05; **p < 0.01). Mean ± SEM is noted. PV: peri-ventricular; LF: left-frontal; RF: ri.ht-frontal; LP: left-parietal; WMHV: white matter hyperintensity volume.

A comparison between AL categories revealed significant differences in PV WMHV between high and no AL risk, and between high and low AL risk individuals (Fig. 2a). Significant differences were also found in LF, RF and LP WMHV, between high and low AL categories (Fig. 2b-d, respectively). No significant differences were observed among AL categories in LT WMHV. Results for Kruskal–Wallis tests and post-hoc Dunn's test for multiple comparisons are detailed in Supplementary Table S4.

For a final confirmation of the potential superiority of ALCS, WMHV showing strong associations with AL in the univariate model (Total, PV, LF, RF, LP and LT) were regressed on AL categories generated by ALES. Overall, fit criteria measured as differences in BIC and R^2^ values favoured the ALCS algorithm, except for LT and LP where no differences were found (Supplementary Table 5).

As WM disease is considered a biomarker of vascular burden, we evaluated if the associations were driven by the cardiovascular component of the AL index. Following the ALCS algorithm, new risk categories were derived from an AL score excluding the cardiovascular biomarkers, and univariate regressions were conducted on Total, PV, LF, RF, LP and LT WMHV. Surprisingly, all associations were lost with the non-cardiovascular AL risk categories, except for Left-frontal WMHV β = 0.098, *p* = 0.019 (Supplementary Table S6).

## Discussion

In this study, we developed a comprehensive AL scoring algorithm (ALCS) by integrating three commonly used approaches: quartiles from sex-specific distributions, clinical thresholds, and medication treatments. Sex-specific distribution of total scores were used to create AL risk categories and compared to those generated by the classic empirical distribution method (ALES).

Ratios of correct classification into AL categories showed no significant differences although low-risk category appeared to be best ranked by the ALCS, whereas medium-risk was over categorized by ALES, suggesting that inclusion of clinical thresholds could increase the categorization sensitivity for mild cases. Both algorithms were highly correlated with age and, although no significant differences were found between scoring, ALCS showed better model fit criteria. This was further confirmed by comparing fit criteria of both algorithms regressed on selected MRI measurements. Overall, the higher sensitivity of ALSC seems to rely on the inclusion of clinical thresholds to score biomarkers above pathological ranges as high-risk. The availability of such values in common clinical settings could provide practitioners a quick overview of AL status of a patient without the need of a large sample to calculate quartiles.

No associations were found between AL categories and educational level, employment status, smoking history, parental history of dementia, direct relatives with AD, APOEe4 carrier status, or subjective memory complaints. However, according to previous evidence, AL risk was strongly predicted by sex and age, with low/medium categories associated to males and medium–high predicted by aging.

Evaluation of AL categories and brain imaging revealed positive associations between higher AL and total, periventricular, right and left frontal and left parietal WMH volumes, and the significant differences across AL categories suggests a dose-dependent response. As a biomarker of cerebrovascular disease^18^, such differences point to the presence of cardiovascular burden, as most findings in WHMV lost association when the cardiovascular component was removed from the AL index. Previous evidence reports inverse associations of AL with total brain and white-matter volumes^15^, as well as with grey-matter density^16,17^. Nonetheless, the populations in these studies were older (72.5 years, SD = 0.7; 69.6 years, SD = 5.2; 72.7 years, SD = 0.7, respectively) than ours and the AL score was derived through the classic empirical approach, making our findings with ALSC in a younger cohort potentially novel to the existing evidence.

Altogether, our results suggest that sustained stress exposure may enhance brain deterioration in mid-life adults and could accelerate later cognitive decline and dementia development. Since regular follow-up assessments are planned in the PREVENT cohort, we expect to evaluate this hypothesis in a prospective study.

A major limitation to derive an AL index is the lack of consensus about criteria for scoring algorithms. The biomarkers, usually measured in a continuous scale, are often categorized, and then summed into a final score. Category thresholds must then be set but, so far, the method to define them has relied almost exclusively on the subjective criteria of researchers. In 2016, a review of 21 studies form National Health and Nutrition Examination Survey (NHANES)^19^ reported 18 different equations and 5 methods for AL scoring, most of them based on the classical quartile approach, others based on clinical guidelines, one merging both criteria, some considering current medications and others not. Overall, evidence favours more comprehensive approaches that includes clinical criteria^5,6^, sex^7,8^, ethnicity^6,20^ and even geographical diversity^21^.

The heterogeneity of algorithms for AL scoring also implies limitations for replicability. In analyses where a predictor is constructed by imprecise criteria, but the predicted variables are widely agreed measurements—as for the case of MRI volumes—it would not be rare to obtain inconsistent results. Moreover, the lack of methodological agreement between studies could mislead conclusions regarding the association of AL on a given disease and limit the comparison of results, even when obtained in similar populations. We expect this study will contribute to achieve an agreed and validated criteria for constructing AL scores that allow more accurate comparisons and replications.

## Methods

### Participants

Data from the PREVENT study (v700 baseline dataset^22^) were used. As described previously^23,24^, the PREVENT cohort recruited mid-life participants (age: 40–59 years) from sites in Edinburgh, West London, Dublin, Cambridge and Oxford, with half reporting a first-degree family history of dementia. All participants provided written informed consent prior to participation.

Ethical approval was granted by the London-Camberwell St Giles National Health Service (NHS) Research Ethics Committee (REC reference: 12/LO/1023, IRAS project ID: 88938), which operates according to the Helsinki Declaration of 1975 (and as revised in 1983) and by Trinity College Dublin School of Psychology Research Ethics Committee (SPREC022021-010) and the St James Hospital/Tallaght University Hospital Joint Research Ethics Committee.

From the 620 participants selected with complete data for AL scoring, 571 had suitable structural MRI at baseline for assessment, 481 had data for the CA1 hippocampal subfield, and 566 for WMH analysis.

### AL scorings

#### Biomarkers collection

Blood samples were collected consent in a fasted state during baseline visit and analysed in local laboratories. Fourteen biomarkers were assessed for inflammatory/immune (creatinine, albumin, C-reactive protein (CRP), fibrinogen), cardiovascular (systolic blood pressure (SBP), diastolic blood pressure (DBP), resting-heart-rate (RHR), and waist-to-hip ratio (WHR)), and metabolic (total cholesterol, high-density-lipoprotein (HDL) cholesterol, low-density-lipoprotein (LDL) cholesterol, glycemia, triglycerides, and body-mass index (BMI)) systems.

#### Comprehensive AL score (ALCS)

Initial categories for “no-risk” (zero points), “at-risk” (one point), and “high-risk” (two points) were defined for each biomarker, based on both clinical thresholds^7,8^ and quartiles from sex-specific distributions. When clinical upper limit (clinical-up) was higher than the 75th percentile (p75: creatinine, triglycerides, CRP, SBP, DBP), at-risk category was defined between ≥ p75–≤ clinical-up (no-risk: < p75 and high-risk: > clinical-up). When clinical upper limit was lower than p75 (total cholesterol, LDL cholesterol, BMI, WHR), at-risk was defined between ≥ clinical-up–≤ p75 (no-risk: < clinical-up, high-risk: > p75). For reverse biomarkers (albumin, HDL cholesterol), if clinical lower limit (clinical-low) was below the 25th percentile (p25), at-risk was defined as ≤ clinical-low–≥ p25 (no-risk: > p25 and high-risk: < clinical-low). For RHR, only clinical categories provided by the British Cardiovascular Society for age and gender were used. Clinical thresholds and quartiles values are detailed in Supplementary Table S7.

Medication treatments coded through the Anatomic Therapeutic Chemical (ATC) classification system^25^ were scored as high-risk as could potentially mask some biomarkers values, as follows: total cholesterol, triglycerides and LDL for lipid modifying agents (C10); systolic and diastolic blood pressure for anti-hypertensive medication (C02, C03, C09); resting heart rate for beta-blockers (C07) or calcium blockers (C08); and glycemia for insulin or analogues (A10).

After summing the scores, high and low quartiles were calculated from sex-specific distributions of total scores ≥ 1, to generate the following final AL risk categories: No-risk: 0 points; Low-risk: 1 point–≤ p25; Medium-risk: > p25–< p75; High-risk: ≥ p75. The decision algorithm is detailed in Fig. 1a.

#### Empirical AL score (ALES)

Following the classical approach^4^, a purely quartile-based AL index was derived from sex-specific distributions to compare against the ALCS. Biomarkers were awarded 1 point if their value was ≥ p75, or ≤ p25 for albumin and HDL cholesterol. After scoring for medications, AL risk categories were generated from total scores by the same method used in ALCS. The decision algorithm is detailed in Fig. 1b.

### Image acquisition and analyses

Structural MRI data were collected using 3 T Siemens Magnetic Resonance Imaging (MRI) scanners (specific models: Verio, Prisma, Prisma Fit, Skyra). Image processing was carried used using FreeSurfer (v7.1.0) and the following derived variables were used in this analysis: Cerebral white-matter volume (WM), total grey-matter volume (GM), subcortical grey-matter volume (SCG), left and right hippocampal volumes (lHC and rHC respectively), CA1 hippocampal subfield volume, and mean cortical thickness for left (LH thickness) and right (RH thickness) hemispheres. Full MRI protocol and Freesurfer analysis are described in detail on Ritchie et al.^22^ (preprint) and Dounavi et al.^26^.

To correct for interindividual variations in intracranial volumes, all volumetric variables were adjusted by the residual correction method^27^ of a least-square-derived linear regression between raw volumes and the estimated intracranial volume (eICV), or the estimated total intracranial volume (eTIV). Regressions using the entire data set were performed as suggested previously for studies involving healthy groups^28,29^. Comparisons between male and female raw and adjusted values were performed by a 2-tailed unpaired t-test, with α = 0.05.

White-matter hyperintensities (WMH) lesion maps were obtained using an automated script on the Statistical Parametric Mapping 12 suite (https://www.fil.ion.ucl.ac.uk/spm/) on FLAIR MRI, as previously described^30,31^. WMH were segmented into frontal, parietal, occipital, temporal, deep and periventricular as previously described^31,32^. Lesion masks were visually inspected and manually corrected. WMH volumes (WMHV) were normalised by total intracranial volume (TIV) to account for individual differences in head size ((WMH/TIV) × 100%), followed by cube root transformation due to right-tailed skewness^31,33^.

### Covariates

Demographic covariates included age, sex, years of education, educational level, smoking history, employment status, parental history of dementia, direct relative with Alzheimer's Disease (AD), *APOEε4* genotype status, and subjective memory complaint variables. Additionally, self-reported sleep quality was assessed through the Pittsburgh Sleep Quality Index PSQI questionnaire^34^. As potential protective factor against stress, an index of self-reported resilient attitudes was included through the Connor-Davidson Resilience Scale (CDRS)^35^.

### Statistical analysis

All analysis were conducted using IBM SPSS Statistics v.27.0^36^. Statistical differences between sex for age, years of education, ALCS and ALES raw scores were evaluated by two-sided Mann Whitney-U rank sum test as normality were below the rejection value of < 0.05 (estimated by Shapiro–Wilk test). For model comparison, both ALCS and ALES were analysed separately, including age, sex, and years of education as covariates in Multinomial Logistic Regression (MLR) models, with the No-Risk category used as reference. Overall model fit and model selection was determined by highest Mc Fadden’s pseudo-R^2^^4^, the Bayesian Information Criterion difference (ΔBIC) >  − 10^37^, and highest log-likelihood parameters. Classification tables were used to compare the accuracy of correct classification into each AL category, and differences were assessed through pooled probabilities and z-transformations. Inter-rater reliability between algorithms was evaluated through contingency tables and Cohen’s kappa index.

Additionally, as aging is the strongest predictor of physiological decline, categories generated by both algorithms were correlated against age and Spearman's rho correlation coefficients were compared using Fisher’s z transformation^38^.

After model validation, a MLR was fitted to assess relationships between ALCS categories and age, sex, educational level, employment status, smoking history, dementia risk variables and subjective memory complaint.

To evaluate associations between ALCS categories, brain volume and WMH, univariate, partially adjusted and fully adjusted linear regression models were fitted as follows:

*Model 1* MRI outcome = β0 + β1 AL category + ε.

*Model 2* MRI outcome = β0 + β1 AL category + β2 sex + β3 age + β4 years of education + ε.

*Model 3* MRI outcome = β0 + β1 AL category + β2 sex + β3 age + β4 years of education + β5 PSQI global score + β6 CDRS score + ε.

Selected MRI measurements showing strong associations with AL were further assessed for variations between AL categories by Kruskal–Wallis test for independent groups, followed by a Bonferroni corrected Dunn’s test for multiple comparisons (α = 0.05). These measurements were also regressed on AL categories generated by ALES, and BIC and R^2^ differences from the univariate models were compared for a final algorithm validation.

## Ethics

Multi-site ethical approval was granted by the UK London-Camberwell St Giles National Health Service (NHS) Research Ethics Committee (REC reference: 12/LO/1023, IRAS project ID: 88938), which operates according to the Helsinki Declaration of 1975 (and as revised in 1983). A separate ethical application for Ireland was submitted for the Dublin site, was reviewed and given a favourable opinion by Trinity College Dublin School of Psychology Research Ethics Committee (SPREC022021-010) and the St James Hospital/Tallaght University Hospital Joint Research Ethics Committee. All substantial protocol amendments have been reviewed by the same ethics committees and favourable opinion was granted before implementation at sites.

All necessary patient/participant consent has been obtained before assessments and the appropriate institutional forms have been archived. Any patient/participant/sample identifiers included were not known to anyone (e.g., hospital staff, patients, or participants themselves) outside the research group so cannot be used to identify individuals.

### Supplementary Information

Supplementary Information.

## Data Availability

Data used in this study is available open access at no cost on the study website (www.preventdementia.co.uk) or via the Alzheimer’s Disease Data Initiative (ADDI) platform (https://doi.org/10.34688/PREVENTMAIN_BASELINE_700V1) and Dementia Platforms UK (DPUK) platform, pending approval of the data access request from the PREVENT steering group committee. For data access requests or guidance for guidance on how to access to data on the above websites, please contact Katie Wells (katie.wells@ed.ac.uk), the National Research Coordinator for the PREVENT dementia Programme.
