# Supplementary material for: Comprehensive allostatic load risk index is associated with increased frontal and left parietal white matter hyperintensities in mid-life cognitively healthy adults
Source: Sci Rep. 2024 Jan 5;14:573. doi: 10.1038/s41598-023-49656-3 (PMC10766612; doi:10.1038/s41598-023-49656-3)
Supplement: Supplementary file 1 — Supplementary Information. [file 41598_2023_49656_MOESM1_ESM.pdf]

Comprehensive allostatic load risk index is associated with increased frontal and left parietal white matter hyperintensities in mid-life healthy cognitively adults.

Ingrid Buller-Peralta[a*], Sarah Gregory[a], Audrey Low[b], Maria-Eleni Dounavi[b], Katie Bridgeman[a], Georgios Ntailianis[a], Brian Lawlor[c,d], Lorina Naci[c,d], Ivan Koychev[e], Paresh Malhotra[f], John T O'Brien[b], Craig W Ritchie[a,g], Graciela Muniz-Terrera[a,h].

[a] Edinburgh Dementia Prevention, Centre for Clinical Brain Sciences, University of Edinburgh, Edinburgh, UK. Outpatients Department Level 2 Western General Hospital, Crewe Rd S, Edinburgh, EH4 2XU, UK.

[b] Department of Psychiatry, School of Clinical Medicine, University of Cambridge, Cambridge, UK. Level E4, Box 189, Addenbrooke's Hospital, Cambridge, CB2 0QQ, UK

[c] Trinity College Institute of Neuroscience, School of Psychology, Trinity College Dublin, Dublin, Ireland. Aras an Phiarsaigh, Trinity College Dublin, Dublin 2, Ireland.

[d] Global Brain Health Institute, Trinity College Dublin, Dublin, Ireland. GBHI Office Room 0.60, Lloyd Building Trinity College Dublin, Dublin 2, Ireland.

[e] Department of Psychiatry, Oxford University, Oxford, UK. Warneford Hospital, Warneford Ln, Headington, Oxford, OX3 7JX, UK.

[f] Department of Brain Sciences, Imperial College London, London, UK. Burlington Danes, The Hammersmith Hospital, Du Cane Road, London, W12 0NN, UK.

[g] Scottish Brain Sciences, Edinburgh, UK. Gyleview House, 3 Redheughs Rigg, South Gyle, Edinburgh, EH12 9DQ, UK.

[h] Ohio University Heritage College of Osteopathic Medicine, Ohio, USA. 191 W Union St, Athens, OH 45701, USA.

Corresponding Author: Ingrid Buller-Peralta (ingrid.buller@ed.ac.uk). Edinburgh Dementia Prevention - Centre for Clinical Brain Sciences, The University of Edinburgh. Outpatients Department Level 2 Western General Hospital, Crewe Rd S, Edinburgh, EH4 2XU, UK.

Supplementary Material

- **Supplementary Table S1.** Statistical comparisons between males and females for age
- **Supplementary Table S2.** Multinomial Logistic Regression for Comprehensive AL scoring (ALSC) risk categories and demographic covariates
- **Supplementary Table S3.** Intracranial and brain volumes before and after eICV or eTIV correction method
- **Supplementary Table S4.** Multiple comparison test of associated MRI measurements between ALCS risk categories
- **Supplementary Table S5.** Model fit comparison between ALCS and ALES algorithms in selected MRI measurements
- **Supplementary Table S6.** Univariate regression analysis of strongly associated WMHV and AL risk categories derived from a non-cardiovascular index.
- **Supplementary Table S7.** Cut-off thresholds for Allostatic Load scorings

Supplementary Table S1. Statistical comparisons between males and females

		Mean ± SD	Normality[a]	Independent samples test[b]
**Age**	Males	51.71 ± 5.56	W=0.931, p<0.001	U=41731.5, p=0.07
	Females	50.97 ± 5.41	W=0.951, p<0.001	
**Years Education**	Males	16.35 ± 3.2	W=0.959, p<0.001	U=47973, p=0.287
	Females	16.75 ± 3.68	W=0.957, p<0.001	
**ALCS score**	Males	6.88 ± 3.99	W=0.939, p<0.001	U=38615.5, p=0.001
	Females	5.79 ± 3.83	W=0.945, p<0.001	
**ALES score**	Males	4.22 ± 2.73	W=0.942, p<0.001	U=41374.5, p=0.047
	Females	3.77 ± 2.64	W=0.939, p<0.001	

[a] Shapiro-Wilk test
[b] 2-sided Mann-Whitney test (α=0.05)

Supplementary Table S2. MLR for ALCS risk categories and demographic covariates
(No-risk category set as reference category)

	**Low risk**		**Medium risk**		**High risk**		Overall likelihood ratio test	
	aOR	95% CI	aOR	95% CI	aOR	95% CI	χ^2 (3)	p
**Age**	1.1	[0.98, 1.23]	1.16 **	[1.04, 1.29]	1.2 **	[1.07, 1.34]	24.66	<0.001
**Sex**	0.11 *	[0.01, 0.86]	0.11 *	[0.01, 0.84]	0.13	[0.02, 1.08]	9.05	0.029
**Educational level**	0.76	[0.44, 1.31]	0.72	[0.42, 1.24]	0.58	[0.33, 1]	9.89	0.019
**Employment**	0.92	[0.1, 8.24]	0.7	[0.08, 6.12]	0.78	[0.09, 6.9]	0.84	0.84
**Smoking history**	0.61	[0.25, 1.5]	0.49	[0.2, 1.2]	0.7	[0.29, 1.73]	6.03	0.11
**Parent dementia history**	5.51	[0.66, 45.99]	5.44	[0.65, 45.53]	3.52	[0.42, 29.72]	7.24	0.065
**Direct relative AD**	0.14	[0.01, 1.37]	0.17	[0.02, 1.64]	0.22	[0.02, 2.12]	4.98	0.173
**APOEε4 carriers**	0.8	[0.25, 2.62]	0.9	[0.28, 2.93]	1.08	[0.33, 3.56]	1.79	0.618
**Subjective memory complaint**	0.76	[0.21, 2.76]	0.67	[0.18, 2.42]	0.7	[0.19, 2.56]	0.59	0.898

aOR: adjusted odds ratio. AD: Alzheimer's disease.

*p<0.05, **p<0.01, *** p<0.001

Supplementary Table S3. Intracranial and brain volumes before and after eICV or eTIV correction method

	Total Sample (n=571)	Males (n=227)	Females (n=344)	p
**eICV (SD)**	1492077.4 (162263.66)	1608918.24 (134196.86)	1414976.03 (129968.82)	<0.001
**WM (SD)**	467785.8 (58412.4)	507380.56 (55512.14)	441657.86 (43788.77)	<0.001
**GM (SD)**	647551.3 (58440.87)	692394.57 (48926.21)	617959.96 (43466.64)	<0.001
**SCG (SD)**	57160.59 (5161.1)	60592.4 (4562.33)	54896 (4196.8)	<0.001
**lHC (SD)**	4018.53 (405.21)	4219.14 (410.02)	3886.15 (343.33)	<0.001
**rHC (SD)**	4136.03 (434.92)	4338.52 (436.41)	4002.41 (379.22)	<0.001
**adj WM (SD)**	467785.8 (34491.36)	473379.87 (36422.7)	464094.36 (32687.99)	0.002
**adj GM (SD)**	647551.3 (35613.36)	658978.09 (33597.12)	640010.95 (34935.93)	<0.001
**adj SC Grey (SD)**	57160.59 (3531.69)	57905.06 (3491.43)	56669.33 (3476.58)	<0.001
**adj lHC (SD)**	4018.53 (326.37)	3985.46 (341.85)	4040.36 (314.34)	0.049
**adj rHC (SD)**	4136.03 (348.46)	4104.83 (361.8)	4156.62 (338.33)	0.082
	**Total Sample (n=481)**	**Males (n=190)**	**Females (n=291)**	**p**
**eTIV (SD)**	1496126.75 (150288.05)	1605755.79 (129132.38)	1424547.65 (116195.93)	<0.001
**CA1**	1371.22 (150.02)	1442.63 (153.47)	1324.6 (127.99)	<0.001
**adj CA1**	1371.22 (134.51)	1333 (133.53)	1396.18 (129.39)	<0.001

p<0.05, statistically significant after 2-tailed unpaired t-test between sex. SD - Standard-deviation. eICV: estimated intracranial volume; WM: white matter volume GM: grey matter volume; SCG: subcortical grey matter volume; lHC: left Hippocampal volume; rHC: right hippocampal volume; eTIV: estimated total intracranial volume (for CA1 correction); CA1: Hippocampal CA1 subregion volume; adj: volume after residual correction method.

Supplementary Table S4. Multiple comparison test of associated MRI measurements between ALCS risk categories

	Kruskal-Wallis test				Dunn's test for multiple comparisons[a]		
	**H(3)**	**p**	**AL category**	**Mean ± SEM**	**vs. Low**	**vs. Medium**	**vs. High**
**PV WMHV[c]**	H(3) = 18.86	<0.001	No risk	1.44 ± 0.84	Q= 1.25; p= 1.000	Q= 1.95; p= 0.308	Q= 2.64; **p= 0.049**
			Low risk	1.2 ± 0.12		Q= 2.02; p= 0.259	Q= 3.87; **p= 0.001**
			Medium risk	1.45 ± 0.13			Q= 2.03; p= 0.253
			High risk	2.12 ± 0.28			
**LF WMHV[c]**	H(3) = 16.65	<0.001	No risk	0.27 ± 0.07	Q= 0.75; p= 1.000	Q= 1.42; p= 0.938	Q= 2.12; p= 0.202
			Low risk	0.39 ± 0.04		Q= 1.94; p= 0.314	Q= 3.82; **p= 0.001**
			Medium risk	0.54 ± 0.06			Q= 2.06; p= 0.238
			High risk	0.8 ± 0.11			
**RF WMHV[c]**	H(3) = 16.33	<0.001	No risk	0.37 ± 0.12	Q= 0.81; p= 1.000	Q= 1.66; p= 0.586	Q= 2.12; p= 0.202
			Low risk	0.45 ± 0.04		Q= 2.47; p= 0.081	Q= 3.66; **p= 0.001**
			Medium risk	0.69 ± 0.12			Q= 1.38; p= 1.000
			High risk	0.83 ± 0.1			
**LP WMHV[c]**	H(3) = 10.05	0.018	No risk	0.05 ± 0.03	Q= 0.11; p= 1.000	Q= 0.73; p= 1.000	Q= 1.23; p= 1.000
			Low risk	0.12 ± 0.03		Q= 1.79; p= 0.443	Q= 3.08; p= **0.012**
			Medium risk	0.15 ± 0.03			Q= 1.45; p= 0.89
			High risk	0.23 ± 0.06			
**LT WMHV[c]**	H(3) = 6.11	0.106	No risk	0.04 ± 0.03			
			Low risk	0.08 ± 0.01			
			Medium risk	0.1 ± 0.02			
			High risk	0.13 ± 0.03			

SCGM: subcortical grey matter volume; WMHV: white matter hyperintensity volume; PV: peri-ventricular; LF: left-frontal; RF: right-frontal; LP: left-parietal; LT: left-temporal.

[a] p values adjusted by Bonferroni correction.

[b] Adjusted to eICV by residual correction method.

[c] Normalized to SPM12 total intracranial volume (TIV).

Supplementary Table S5. Model fit comparison between ALCS and ALES algorithms in selected MRI measurements[a]

		F(1,565)	R^2	ΔR^2	BIC	ΔBIC
**Total WMHV**	ALCS	7.68**	0.013	0.001	-61.6553	-0.6929
	ALES	6.99**	0.012	REF	-60.9624	REF
**PV WMHV**	ALCS	10.54**	0.018	0.003	-616.0605	-2.048
	ALES	8.46**	0.015	REF	-614.0125	REF
**LF WMHV**	ALCS	17.06***	0.029	0.009	-1712.238	-5.3112
	ALES	11.44**	0.02	REF	-1706.927	REF
**RF WMHV**	ALCS	8.04**	0.014	0.002	-1373.027	-1.366
	ALES	6.66*	0.012	REF	-1371.661	REF
**LP WMHV**	ALCS	4.2*	0.007	-0.001	-2323.829	0
	ALES	4.32*	0.008	REF	-2323.829	REF
**LT WMHV**	ALCS	3.97*	0.007	0	-3048.837	0
	ALES	3.72	0.007	REF	-3048.837	REF

WMHV: white matter hyperintensity volume, PV: peri-ventricular; LF: left-frontal; RF: right-frontal; LP: left-parietal; LT: left-temporal.

[a] MRI measurements selected for showing strong associations when regressed on ALCS univariate model.

* p < 0.05; ** p < 0.01; *** p < 0.001

Supplementary Table S6. Univariate regression analysis of strongly associated WMHV and AL risk categories derived from a non-cardiovascular index[a]

	F(1,565)	β^b	p
**Total WMHV**	2.58	0.067	0.109
**PV WMHV**	3.5	0.079	0.062
**LF WMHV**	5.49	0.098	0.019*
**RF WMHV**	3.23	0.75	0.073
**LP WMHV**	1.33	0.049	0.249
**LT WMHV**	1.39	0.05	0.239

WMHV: white matter hyperintensity volume, PV: peri-ventricular; LF: left-frontal; RF: right-frontal; LP: left-parietal; LT: left-temporal.

[a] MRI measurements selected for showing strong associations when regressed on ALCS univariate model.

[b] Standardized regression coefficients reported.

* $p < 0.05$

Supplementary Table S7. Cut-off thresholds for Allostatic Load (AL) scorings

System	Biomarker	Gender	Clinical thresholds	p75 (p25*)	Comprehensive Score			Empirical Score	
					No-risk	*At-risk*	*High-risk*	*No-risk*	*At-Risk*
Immune	**Creatinine [µmol/L]**	Male	59 - 104	88.00	≤87.99	≥88 - ≤104	≥104.01	<88	≥87.99
		Female	45 - 84	69.00	≤68.99	≥69 - ≤84	≥84.01	<69	≥68.99
	**Albumin* [gr/L]**	Male	35 - 50	40.00	≥40.01	≤35 - ≥40	≤34.99	>40	≤40.01
		Female	same as Male	38.00	≥38.01	≤35 - ≥38	≤34.99	>38	≤38.01
	**C-reactive protein (CRP) [mg/L]**	Male	<5	3.65	≤3.64	≥3.65 - ≤4.99	≥5	<3.65	≥3.64
		Female	same as Male	4.00	≤3.99	≥4 - ≤4.99	≥5	<4	≥3.99
	**Fibrinogen [gr/L]**	Male	1.7 – 4.98	3.10	≤3.09	≥3.1 - ≤4.98	≥4.99	<3.1	≥3.09
		Female	same as Male	3.38	≤3.37	≥3.38 - ≤4.98	≥4.99	<3.38	≥3.37
Metabolic	**Total Cholesterol [mmol/L]**	Male	0 - 5.0	5.94	≤5	≥5.01 - ≤5.94	≥5.95	<5.94	≥5.93
		Female	same as Male	6.19	≤5	≥5.01 - ≤6.19	≥6.2	<6.19	≥6.18
	**HDL Cholesterol* [mmol/L]**	Male	1.0 – 3.0	1.17	≥1.18	≥1 - ≤1.17	≥0.99	>1.17	≤1.18
		Female	same as Male	1.48	≥1.49	≤1 - ≥1.48	≤0.99	>1.48	≤1.49
	**LDL Cholesterol [mmol/L]**	Male	1.0 – 3.0	3.8	≤3	≤3.01 - ≥3.8	≤3.81	<3.8	≥3.79
		Female	same as Male	3.83	≤3	≥3.01 - ≤3.83	≥3.84	<3.83	≥3.82
	**Triglycerides [mmol/L]**	Male	0 – 1.7	1.6	≤1.59	≥1.6 - ≤1.7	≥1.71	<1.6	≥1.59
		Female	same as Male	1.2	≤1.19	≥1.2 - ≤1.7	≥1.71	<1.2	≥1.19
	**Glycemia [mmol/L]**	Male	5.5 - 6.9	5.4	≤5.39	≥5.4 - ≤6.9	≥6.91	<5.4	≥5.39
		Female	same as Male	5.2	≤5.19	≥5.2 - ≤6.9	≥6.91	<5.2	≥5.19
	**Body mass index (BMI) [kg/m2]**	Male	25 - 29.9	30.33	≤24.99	≥25 - ≤29.99	≥30	<30.33	≥30.32
		Female	same as Male	30.22	≤24.99	≥25 - ≤29.99	≥30	<30.22	≥30.21
Cardiovascular	**Systolic blood pressure (SBP) [mmHg]**	Male	120 - 149	140.5	≤140.49	≥140.5 - ≤149	≥149.01	<140.5	≥140.49
		Female	same as Male	127.67	≤127.66	≥127.67 - ≤149	≥149.01	<127.67	≥127.66
	**Diastolic blood pressure (DBP) [mmHg]**	Male	80 - 89	86.17	≤86.16	≥86.17 - ≤89	≥89.01	<86.17	≥86.16
		Female	same as Male	79	≤78.99	≥79 - ≤89	≥89.01	<79	≥78.99
	**Waist-to-hip ratio (WHR)**	Male	no-risk ≤0.95, risk: 0.96-0.99, high-risk ≥1	0.98	≤95.99	≥96 - ≤0.99	≥1	<0.98	≥0.97
		Female	no-risk ≤0.80, risk: 0.81-0.84, high-risk ≥85	0.87	≤80.99	≥0.81 - ≤0.84	≥85	<0.87	≥0.86
	**Resting heart rate (RHR) [BPM]**								
	Age ≤45	Male	average: <76, below average: 76-82, poor:>82	69.67	≤75.99	≥76 - ≤82	≥82.01	<69.67	≥69.67
		Female	average: <79, below average: 79-84, poor:>84	69.83	≤78.99	≥79 - ≤84	≥84.01	<69.83	≥69.83
	Age 46 - 55	Male	average: <77, below average: 77-83, poor:>83	67	≤76.99	≥77 - ≤83	≥83.01	<67	≥67

		Female	average: <78, below average: 78-83, poor:>83	69.67	≤77.99	≥78 - ≤83	≥83.01	<69.67	≥69.67
	Age 56 - 65	Male	average: <76, below average: 76-81, poor:>81	67	≤75.99	≥76 - ≤81	≥81.01	<68	≥68
		Female	average: <78, below average: 78-83, poor:>83	69.67	≤77.99	≥78 - ≤83	≥83.01	<68.67	≥68.67

* Reverse scorings (p25 or low clinical thresholds used for risk)
